# Supplementary material for: Meta-Analysis and Validation of a Colorectal Cancer Risk Prediction Model Using Deep Sequenced Fecal Metagenomes
Source: Cancers (Basel). 2022 Aug 30;14(17):4214. doi: 10.3390/cancers14174214 (PMC9454621; doi:10.3390/cancers14174214)
Supplement: Supplementary file 1 [file cancers-14-04214-s001.zip › cancers-1877802-supplementary.pdf]

Supporting information

Figure S1. PRISMA chart of the search strategy

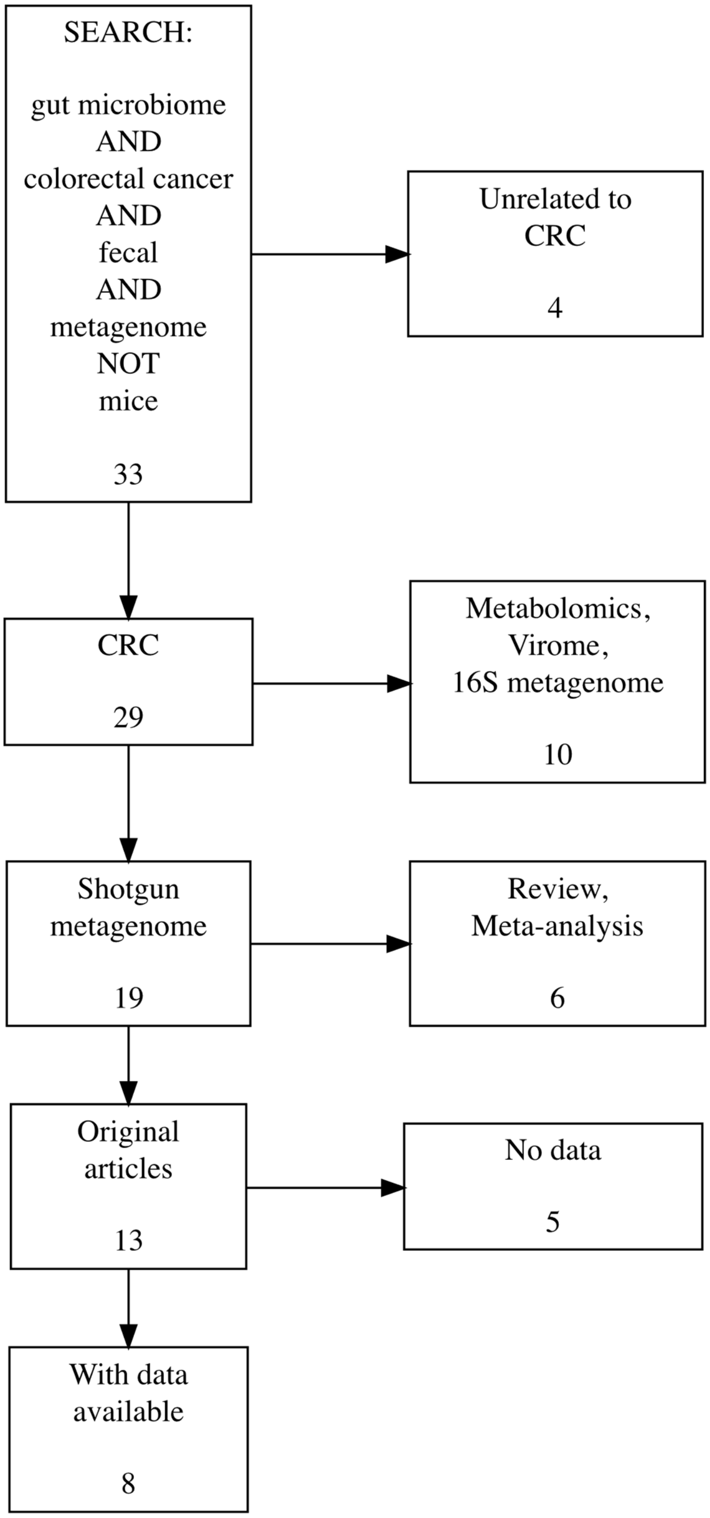

Figure S2. Epidemiological description of the included metagenomic datasets. a) Age distribution of participants. b) Sex distribution of participants. c) BMI distribution of participants.

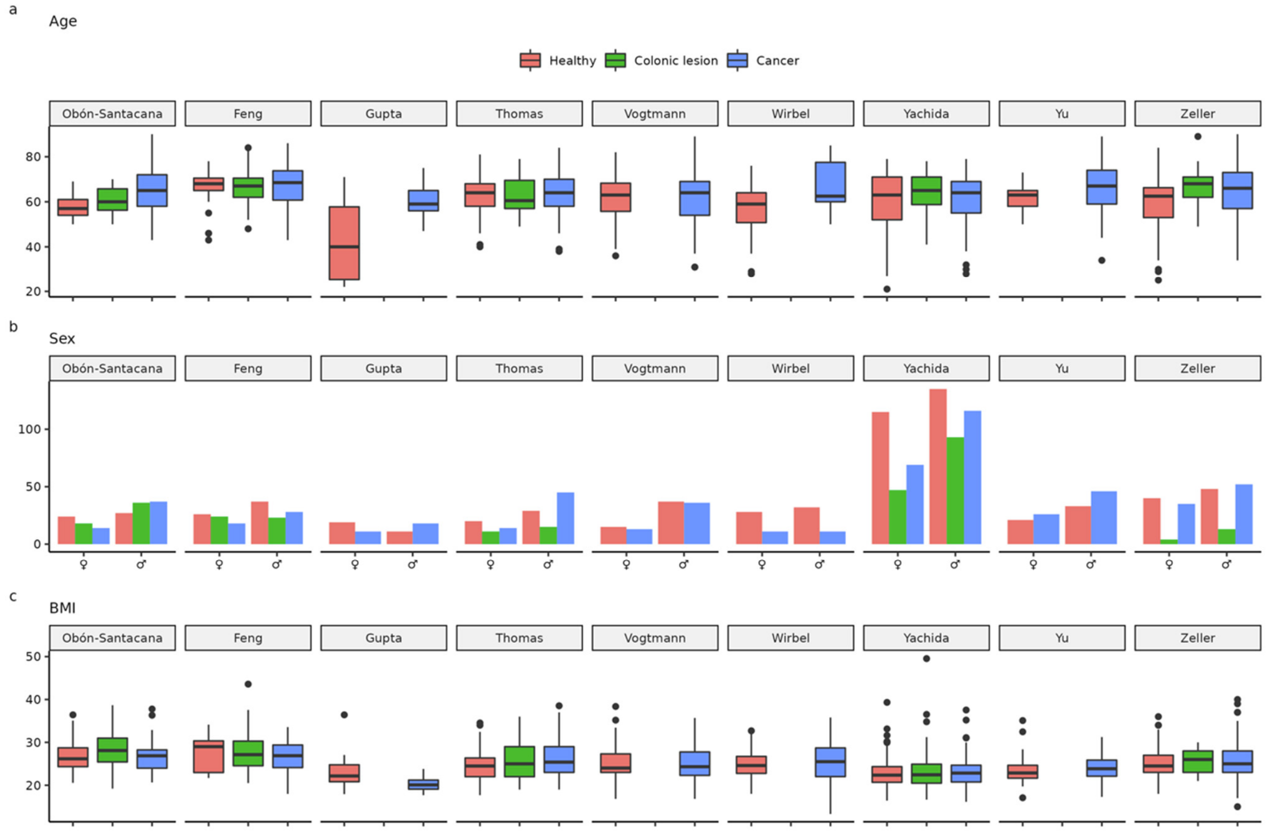

Figure S3. Beta diversity metrics (based on Euclidean distances of ILR-transformed relative abundance counts) of the included metagenomic datasets by health status.

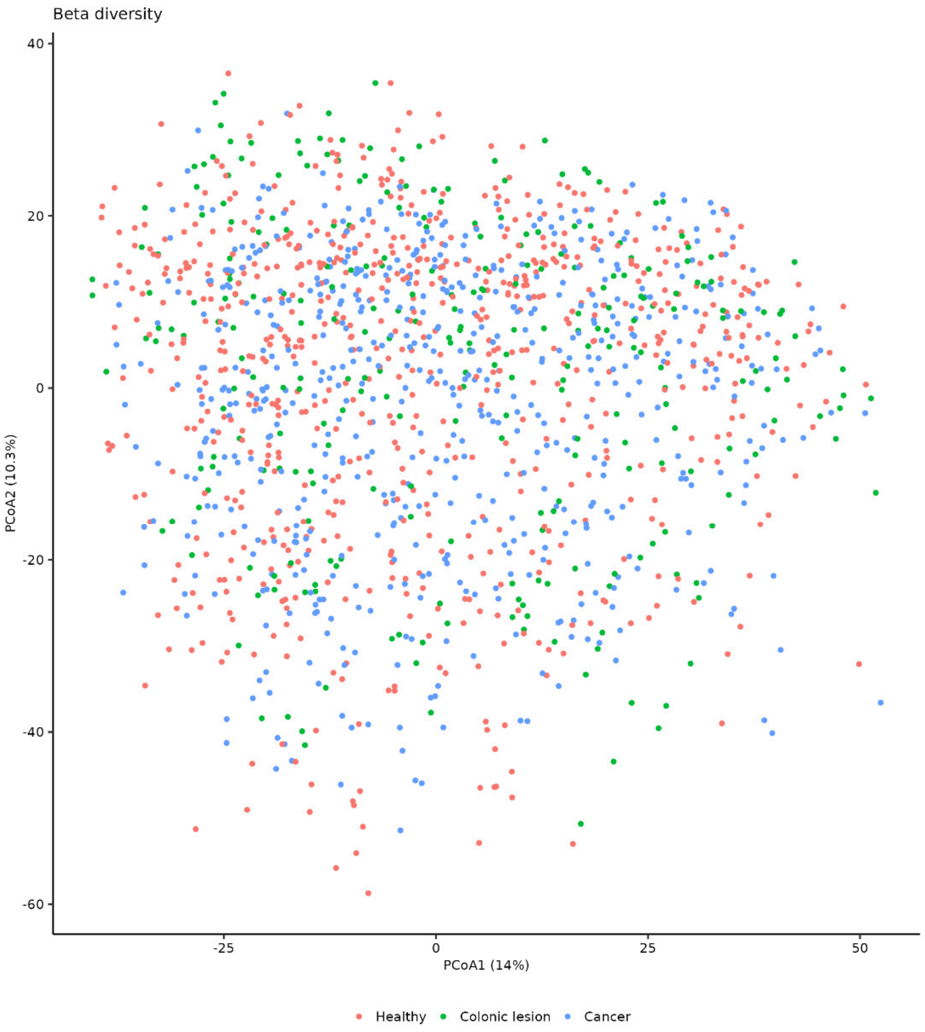

Figure S4. Microbiome diversity statistics of the included metagenomic datasets by age. a) Alpha diversity metrics (Faith's index). b) Beta diversity metrics (based on Euclidean distances of ILR-transformed relative abundance counts). Age median value: 63 years (<63, ≥63).

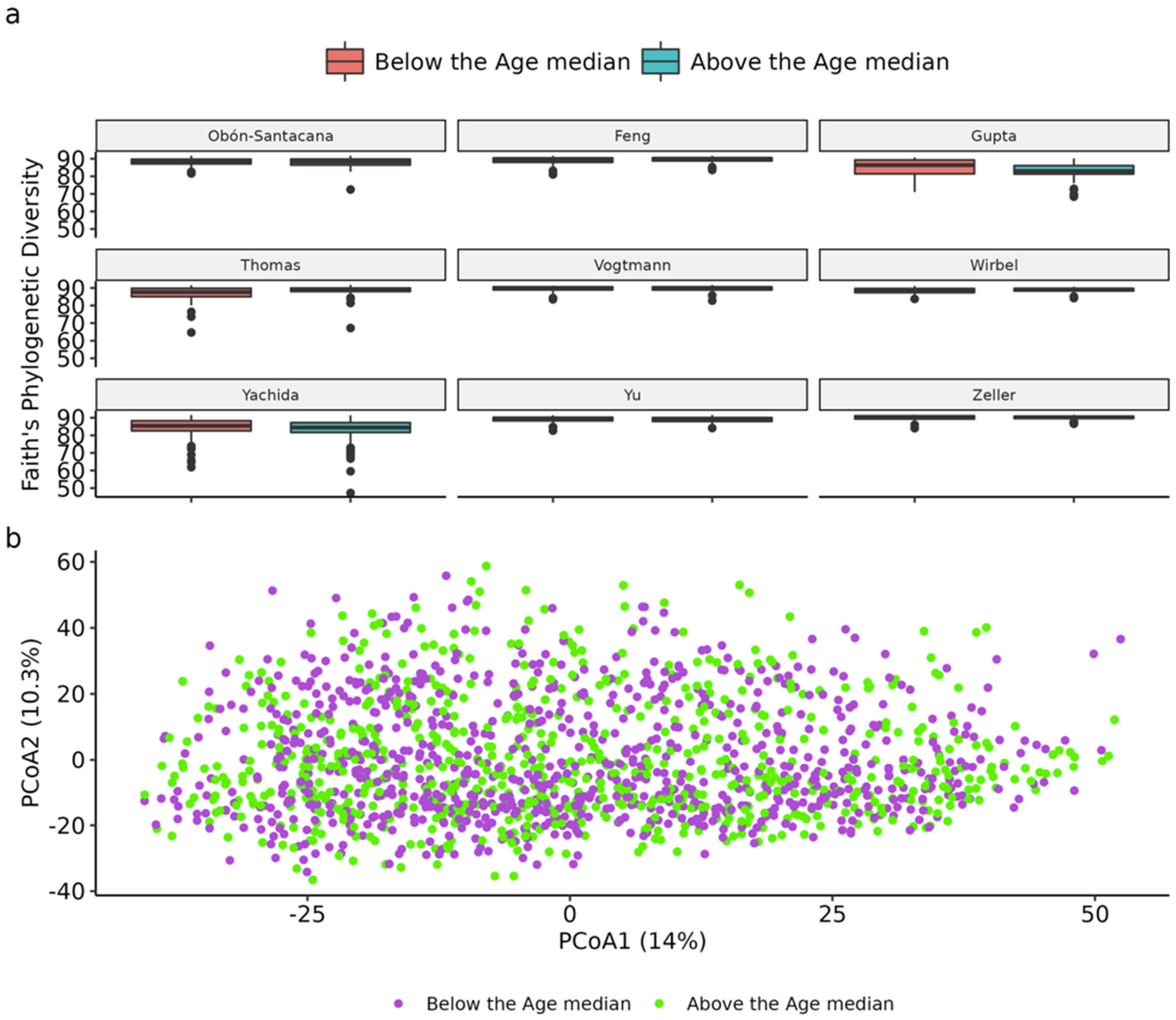

Figure S5. Microbiome diversity statistics of the included metagenomic datasets by sex. a) Alpha diversity metrics (Faith's index). b) Beta diversity metrics (based on Euclidean distances of ILR-transformed relative abundance counts).

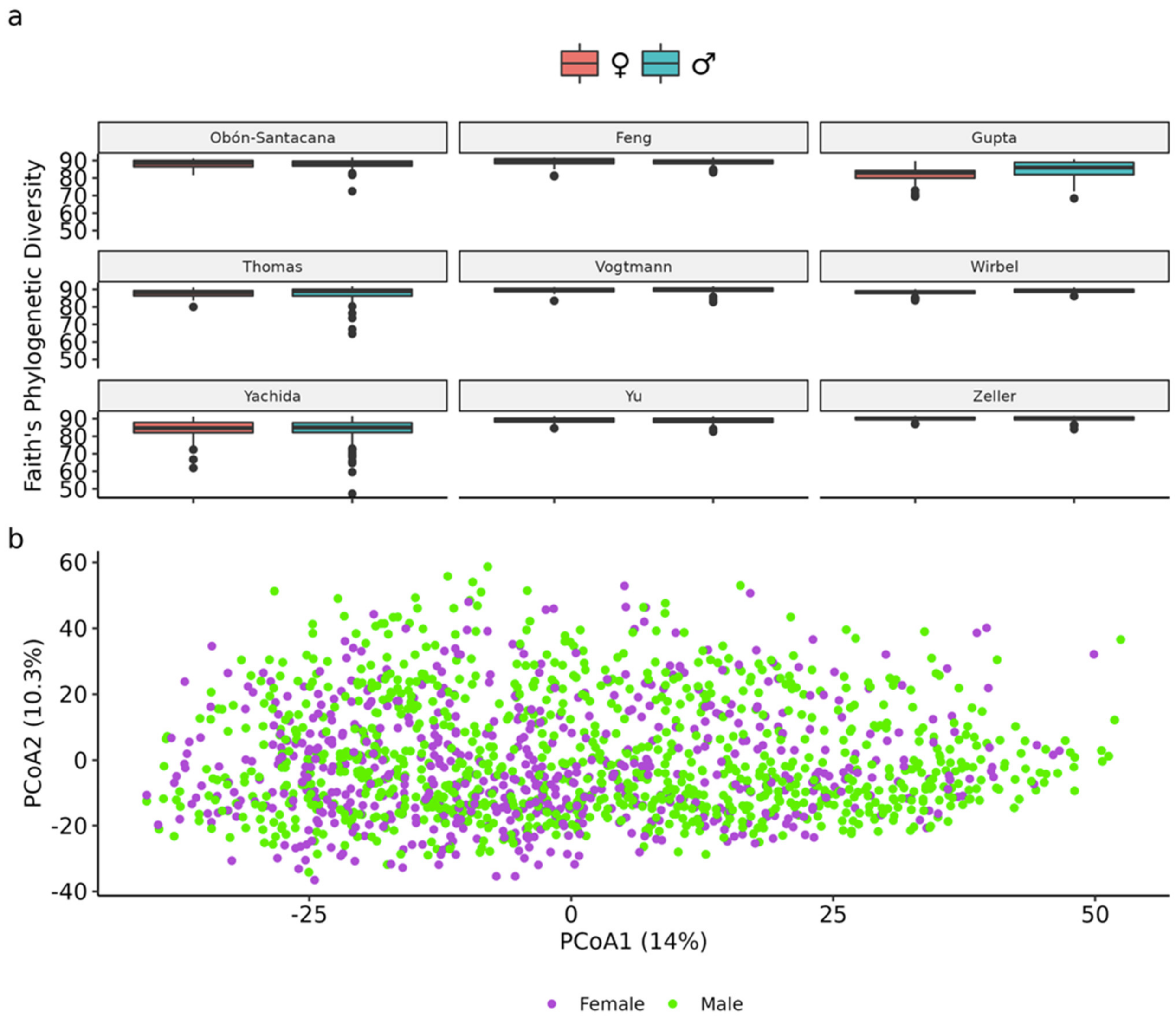

Figure S6 Microbiome diversity statistics of the included metagenomic datasets by body mass index (BMI). a) Alpha diversity metrics (Faith's index). b) Beta diversity metrics (based on Euclidean distances of ILR-transformed relative abundance counts). BMI median value: 24 (<24, ≥24).

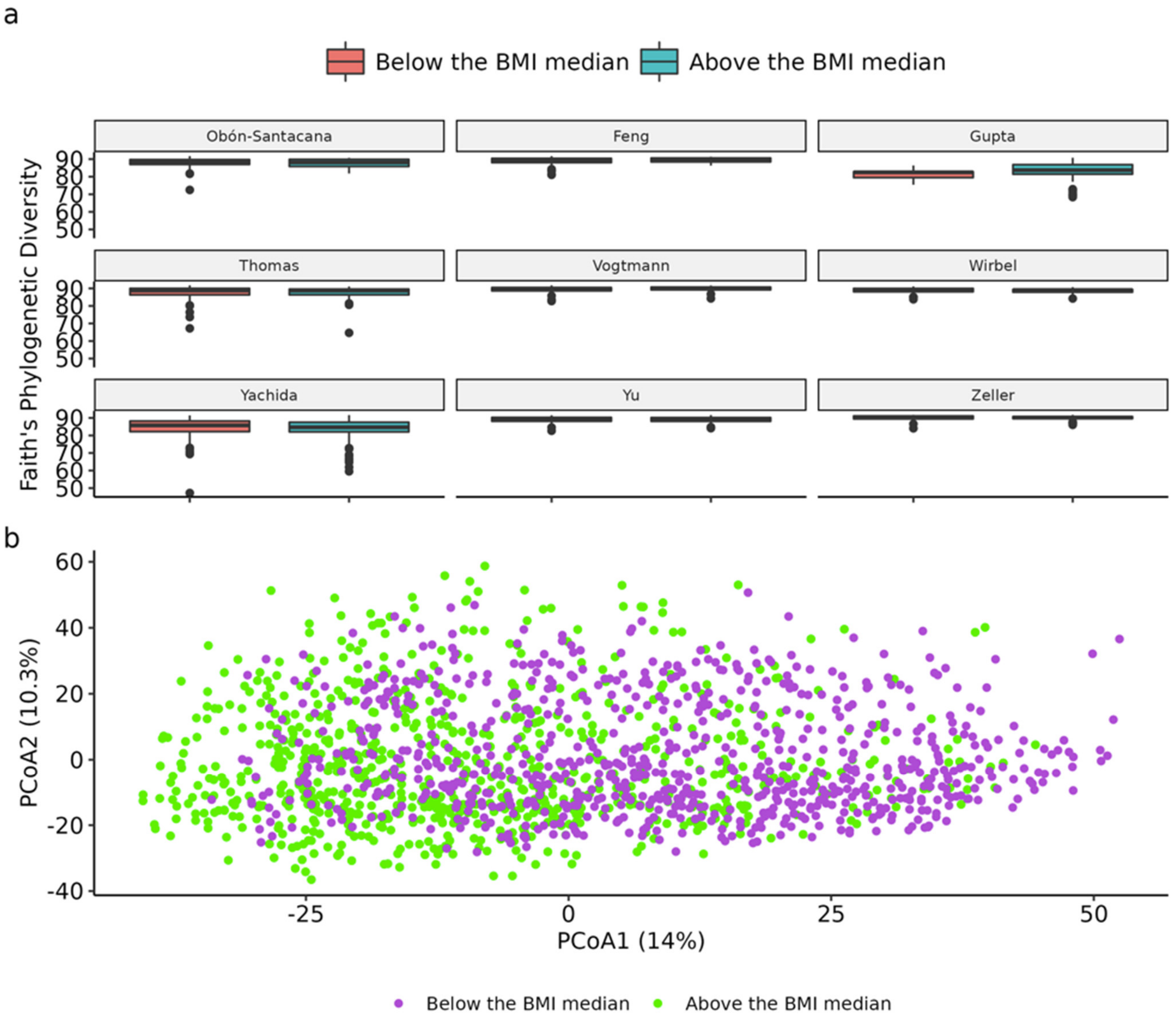

Figure S7. Receiver operating characteristic curve representing the CRC-trained model to predict the presence of precancerous lesions

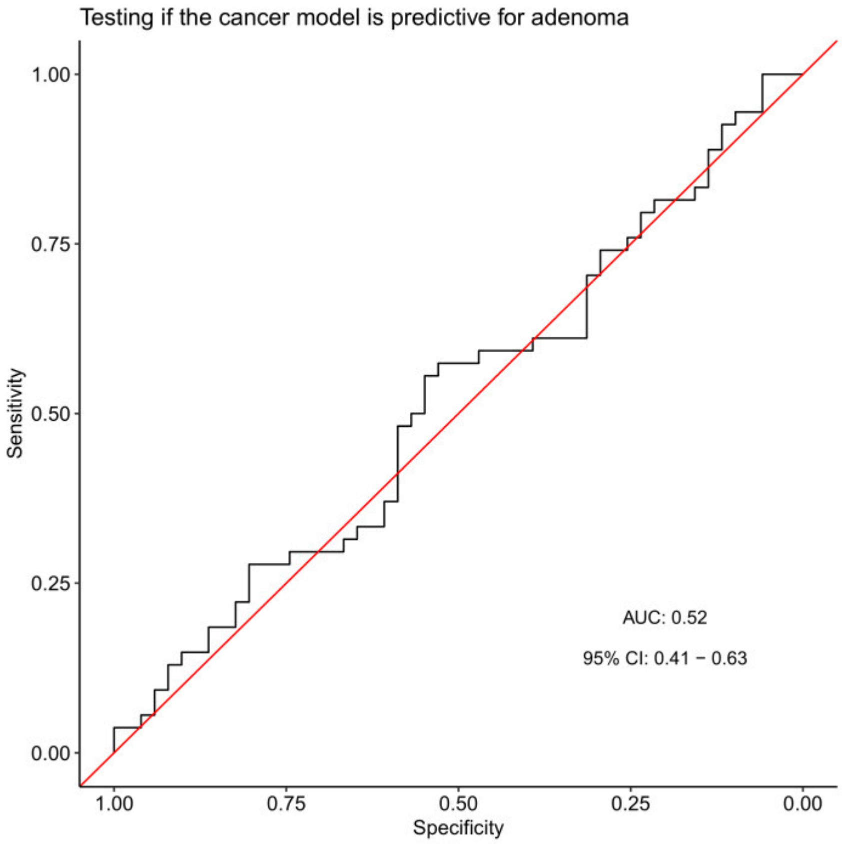

Figure S8. Summary of the LASSO precancerous lesion trained model. a) Log-transformed relative abundance counts of control-enriched (green) and precancerous lesion-enriched (purple) species selected by the model. b) Density plot of model prediction, colored by the status of the samples. c) Receiver operating characteristic curve representing the performance of the model to predict the presence of precancerous lesions considering all species as candidates.

*: statistically significant (p-value < 0.05) based on Wilcoxon rank sum test in *aldex.ttest*

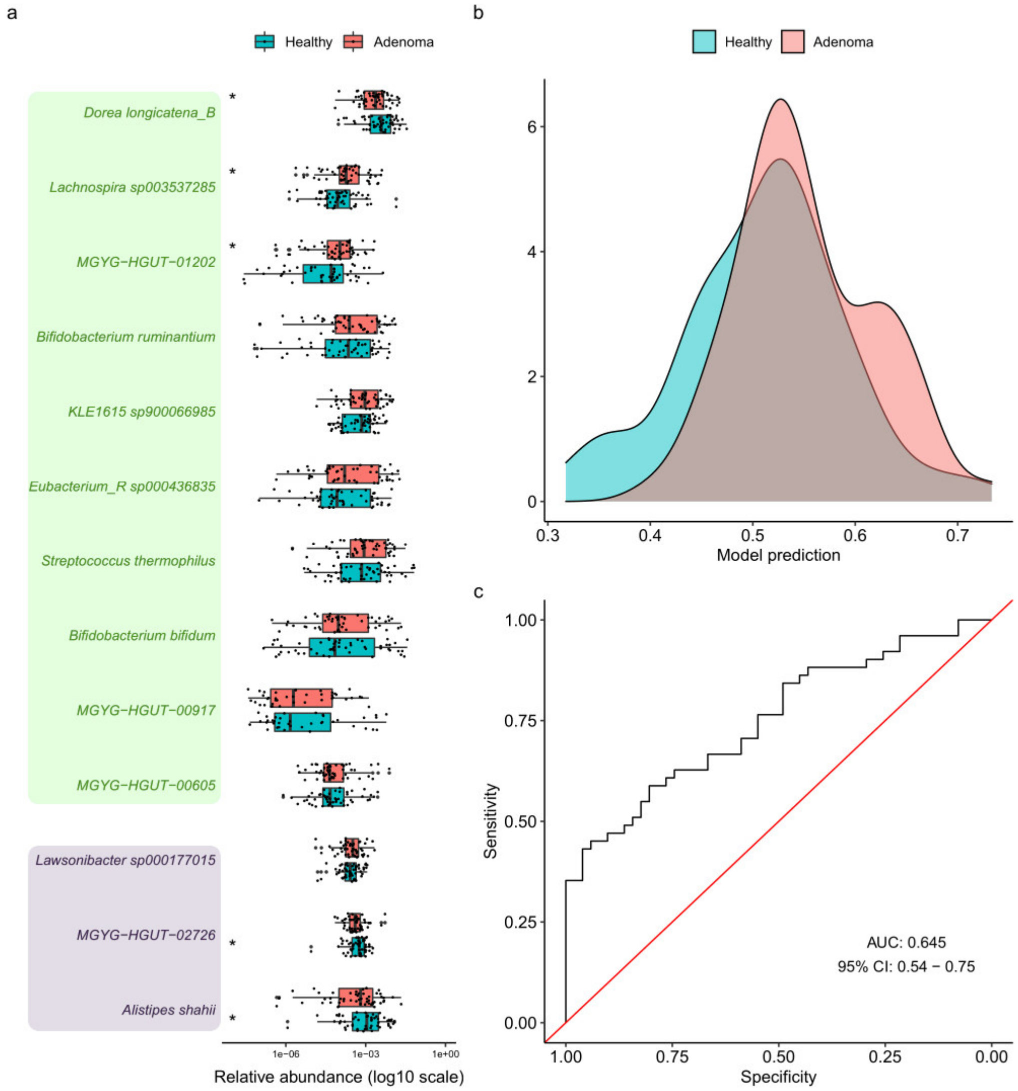

Figure S9. Summary of the LASSO model considering both CRC and precancerous lesion samples and all species. a) Log-transformed relative abundance counts of control-enriched (green) and CRC+precancerous lesion enriched (purple) species selected by the model. b) Density plot of model prediction, colored by the status of the samples. c) Receiver operating characteristic curve representing the performance of the model to predict the presence of CRC and precancerous lesions considering all species as candidates. *: statistically significant (p-value < 0.05) based on Wilcoxon rank sum test in *aldex.ttest*

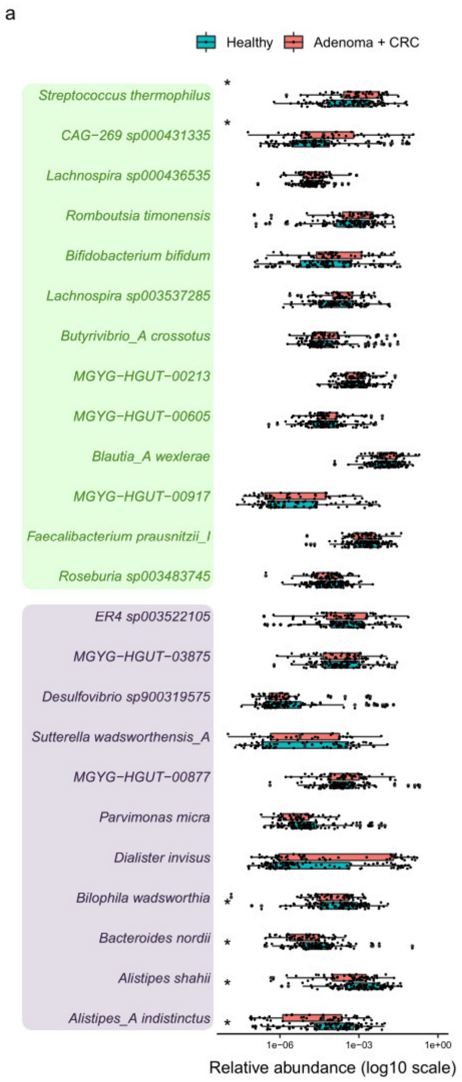

Figure S10. Receiver operating characteristic curve representing the precancerous lesions trained model to predict the presence of CRC in the analysis of eggNOG orthologous groups.

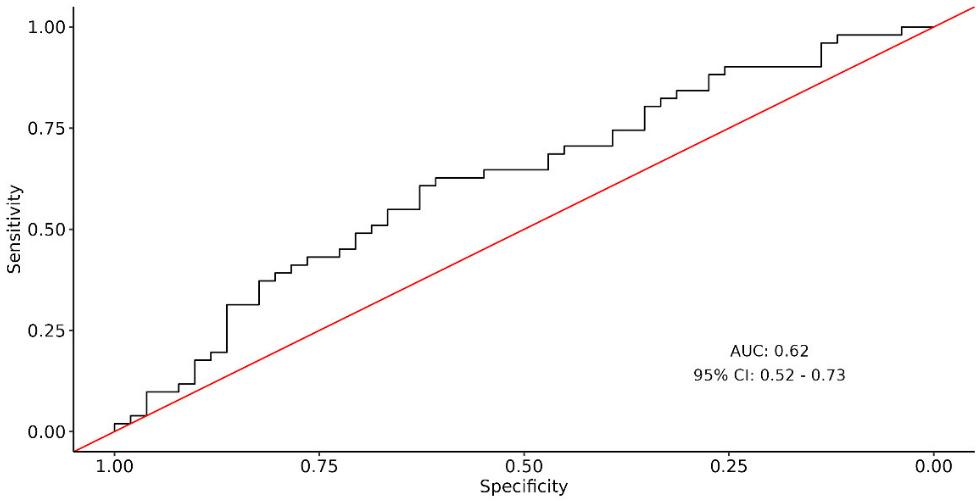

Table S1. Quality control for our 156 COLSCREEN samples. Comparison of the original and high-quality microbial paired-end reads, as well as percentages of read pairs excluded due to duplication or quality and adapter trimming.

Sample	Microbiological reads	High-quality reads	%deduplicated	%trimmed
AL3936	137807640	130385056	0.05	5.34
AL3937	32132994	28949268	3.12	7.01
AL3938	116244474	110262862	0.05	5.10
AL3939	112309536	105909072	0.04	5.66
AL3941	35416308	30577024	2.94	11.05
AL3942	36525444	32121802	3.10	9.25
AL3943	173183254	164643138	0.04	4.89
AL3944	129632482	122562568	0.04	5.41
AL3945	30127354	26587002	3.61	8.45
AL3946	29971510	26600940	3.54	7.99
AL3947	153180552	145616518	0.03	4.90
AL3948	94936560	89717148	0.04	5.46
AL3949	44379690	41680280	0.05	6.04
AL3950	155630786	147701524	0.05	5.05
AL3951	149547548	142064718	0.05	4.96
AL3952	29865278	26208538	3.36	9.19
AL3953	155187520	146917344	0.05	5.28
AL3954	148838472	141130206	0.05	5.13
AL3955	131602532	124281592	0.05	5.52
AL3956	119522738	112726596	0.04	5.64
AL3957	29192338	26921156	0.04	7.74
AL3958	29059372	27237392	0.05	6.23
AL3959	30756680	27504364	3.47	7.36
AL3960	30600070	27692380	3.11	6.60
AL3961	29780872	27348614	0.05	8.12
AL3962	134107242	127562054	0.05	4.83
AL3963	167411282	158991970	0.05	4.98
AL3964	94684244	86587566	3.08	5.65
AL3965	133713908	125825090	0.05	5.86
AL3966	116984080	107793146	2.67	5.33
AL3967	115875504	106261662	3.16	5.30
AL3968	31187232	27919578	3.25	7.47
AL3969	118342352	112023164	0.05	5.29
AL3970	118551076	112517712	0.03	5.06
AL3971	123738728	117279674	0.06	5.17
AL3972	132033844	125052722	0.05	5.24

AL3973	118066474	111019206	0.02	5.95
AL3974	90101028	82347182	3.45	5.34
AL3975	35941418	31378398	2.59	10.38
AL3976	101065154	95421582	0.05	5.54
AL3977	28686052	26671554	0.06	6.97
AL3978	172682872	162672388	0.05	5.75
AL3979	127728836	121080278	0.04	5.16
AL3980	144391082	136630046	0.05	5.33
AL3981	118854808	112260370	0.04	5.51
AL3982	133009574	125902564	0.04	5.30
AL3983	107280800	98545626	2.85	5.44
AL3984	124896186	118063494	0.04	5.43
AL3985	33104848	30885658	0.05	6.66
AL3986	107197440	101123954	0.04	5.63
AL3987	30283018	27178696	3.33	7.16
AL3988	100431040	92122742	2.84	5.59
AL3989	118785176	112198364	0.04	5.50
AL3990	33386680	31046184	0.05	6.96
AL3991	30041974	26846790	2.98	7.89
AL3992	37443762	34809862	0.05	6.99
AL3993	33731150	30197032	3.17	7.54
AL3994	31988038	28896652	2.90	6.96
AL3995	32001504	29654910	0.04	7.29
AL3996	30020518	27716642	0.05	7.63
AL3997	29887790	26858190	3.06	7.30
AL3998	87720900	80362548	2.79	5.76
AL3999	92834350	84224352	3.21	6.26
AL4000	36227656	33343526	0.05	7.92
AL4001	39006180	34900620	3.51	7.27
AL4002	34108148	30677586	3.41	6.88
AL4003	29059002	26823874	0.05	7.65
AL4004	95958410	90723462	0.05	5.41
AL4005	30625786	28247258	0.04	7.73
AL4006	33203256	29830902	3.63	6.77
AL4007	35829114	32347982	3.65	6.29
AL4008	40284218	36460120	3.64	6.08
AL4009	36438206	33575524	0.05	7.81
AL4010	106245442	100296052	0.05	5.55
AL4011	32382424	28585982	3.57	8.45
AL4012	100130168	91499656	3.24	5.56
AL4013	32389742	29332026	3.19	6.46
AL4014	30341970	27090298	3.43	7.55
AL4015	31674774	27944114	3.51	8.57

AL4016	54293154	50882104	0.05	6.24
AL4017	54669778	51436142	0.05	5.87
AL4018	44068616	40941006	0.05	7.05
AL4019	86110594	80928636	0.05	5.97
AL4020	31144764	27172228	3.53	9.57
AL4021	30020926	26531128	3.53	8.39
AL4022	30331004	26790910	3.72	8.26
AL4023	35354818	32607326	0.05	7.72
AL4024	35550064	31714118	3.05	7.99
AL4025	53888168	50315258	0.05	6.59
AL4026	91113432	85028846	0.05	6.64
AL4027	36555904	32477984	3.15	8.26
AL4028	35196730	31490354	3.26	7.52
AL4029	35884498	32274882	3.39	6.91
AL4030	40191780	37296710	0.05	7.16
AL4031	43508306	40521402	0.05	6.82
AL4032	44470096	40084828	3.27	6.81
AL4033	38614780	34870124	3.20	6.71
AL4034	70561786	66300300	0.04	6.00
AL4035	31936526	28410442	2.96	8.33
AL4036	34644488	32139988	0.05	7.18
AL4037	32124472	28559660	2.70	8.63
AL4038	38384538	34457260	3.12	7.34
AL4039	34893322	31228404	3.32	7.43
AL4040	39679232	36822692	0.04	7.16
AL4041	53851648	50350632	0.05	6.45
AL4042	34971506	31286978	2.91	7.85
AL4043	32267220	29463770	0.05	8.65
AL4044	29862710	26520896	3.02	8.42
AL4045	31911920	28237536	3.14	8.64
AL4046	33535138	31142942	0.05	7.09
AL4047	43173862	38853088	3.92	6.34
AL4048	48311318	43965720	2.29	6.86
AL4049	41774472	37244892	3.27	7.82
AL4050	41289386	37373082	3.78	5.93
AL4051	39056812	36254166	0.05	7.13
AL4053	41987652	38914320	0.04	7.28
AL4054	35443942	31692712	2.93	7.88
AL4055	35596278	31688046	3.22	8.02
AL4056	36494028	33494606	0.05	8.17
AL4057	47077818	42684832	3.20	6.34
AL4058	302212934	287890190	0.05	4.69
AL4059	42709396	38469566	3.19	6.96

AL4060	41784252	36966762	3.19	8.62
AL4061	39118816	36622546	0.06	6.33
AL4062	39599020	35846782	2.88	6.79
AL4063	39038374	35885604	0.05	8.03
AL4064	1125476974	1051116174	0.07	6.55
AL4065	47338180	43876110	0.05	7.27
AL4066	42921990	38490450	3.01	7.54
AL4067	43903990	41010182	0.05	6.55
AL4068	41896332	38750650	0.06	7.45
AL4069	45717170	42127050	0.05	7.81
AL4070	45940712	41041760	3.14	7.76
AL4071	52631110	48971764	0.05	6.91
AL4072	43137226	39758256	0.04	7.79
AL4073	2947937736	2749988242	0.09	6.63
AL4074	49244426	44455586	2.88	7.05
AL4075	46725310	43375632	0.05	7.13
AL4076	39362420	36498372	0.05	7.23
AL4077	38600364	35688364	0.05	7.50
AL4078	46367270	43137208	0.05	6.92
AL4079	40686892	37533144	0.05	7.71
AL4080	44195718	39191994	3.01	8.57
AL4081	64619284	60272194	0.05	6.68
AL4082	71013766	66289598	0.06	6.60
AL4083	31698982	29505854	0.06	6.87
AL4084	84621300	75558410	2.95	8.00
AL4086	46819138	43199158	0.05	7.69
AL4087	42681434	39376352	0.05	7.70
AL4088	54539776	50595698	0.05	7.19
AL4089	43757436	40364166	0.06	7.70
AL4090	54095394	49049762	2.90	6.62
AL4091	46816712	43234668	0.05	7.61
AL4092	53819896	48636674	3.59	6.27
AL4093	45069952	41306620	0.05	8.31
AL4095	39516740	36084140	0.04	8.65

Table S2. Detailed description of our 156 COLSCREEN samples.

sampleID	cohort	status	age	sex	bmi	sampling to colonoscopy
AL3936	screening	cancer	57	male	32	before
AL3937	screening	cancer	62	male	26	before
AL3938	screening	cancer	55	female	27	before
AL3939	screening	cancer	57	female	27	before
AL3941	hospital	cancer	58	female	28	after
AL3942	screening	cancer	68	male	36	before
AL3943	hospital	cancer	75	female	33	after
AL3944	screening	cancer	58	male	29	before
AL3945	screening	cancer	60	male	26	before
AL3946	hospital	cancer	60	male	29	after
AL3947	screening	cancer	62	male	29	before
AL3948	hospital	cancer	66	male	27	after
AL3949	hospital	cancer	76	male	27	after
AL3950	hospital	cancer	65	female	24	after
AL3951	hospital	cancer	68	male	23	after
AL3952	hospital	cancer	56	male	33	after
AL3953	screening	adenoma	52	female	28	before
AL3954	screening	adenoma	70	male	26	before
AL3955	screening	adenoma	59	male	30	before
AL3956	screening	adenoma	53	male	26	before
AL3957	screening	adenoma	66	female	28	before
AL3958	screening	adenoma	69	female	32	before
AL3959	screening	adenoma	59	male	25	before
AL3960	screening	adenoma	59	male	31	before
AL3961	screening	adenoma	53	male	31	before
AL3962	screening	adenoma	60	male	25	before
AL3963	screening	adenoma	52	female	24	before
AL3964	screening	adenoma	68	male	30	before
AL3965	screening	adenoma	67	female	25	before
AL3966	screening	neg	55	female	23	before
AL3967	screening	neg	64	male	28	before
AL3968	screening	neg	52	male	33	before
AL3969	screening	neg	69	male	27	before
AL3970	screening	neg	53	female	26	before
AL3971	screening	neg	58	female	29	before
AL3972	screening	neg	65	male	35	before
AL3973	screening	neg	56	male	27	before
AL3974	screening	neg	52	female	24	before
AL3975	screening	neg	59	male	24	before

AL3976	screening	neg	59	male	36	before
AL3977	screening	neg	51	male	22	before
AL3978	screening	neg	62	male	27	before
AL3979	screening	neg	58	female	21	before
AL3980	screening	adenoma	66	male	25	before
AL3981	screening	neg	69	female	28	before
AL3982	screening	adenoma	69	male	38	before
AL3983	screening	adenoma	59	male	30	before
AL3984	screening	neg	59	male	30	before
AL3985	screening	adenoma	66	male	32	before
AL3986	hospital	cancer	60	female	31	after
AL3987	screening	adenoma	61	female	29	before
AL3988	screening	cancer	64	male	24	before
AL3989	screening	adenoma	63	male	28	before
AL3990	screening	neg	61	female	31	before
AL3991	screening	adenoma	67	male	29	before
AL3992	screening	cancer	66	male	27	before
AL3993	screening	neg	65	male	32	before
AL3994	screening	cancer	66	male	23	before
AL3995	screening	adenoma	57	male	33	before
AL3996	screening	adenoma	65	male	37	before
AL3997	screening	adenoma	55	female	29	before
AL3998	screening	cancer	61	female	24	before
AL3999	screening	neg	66	male	28	before
AL4000	screening	adenoma	64	female	38	before
AL4001	screening	adenoma	56	male	27	before
AL4002	hospital	cancer	73	male	22	after
AL4003	screening	adenoma	57	male	28	before
AL4004	screening	adenoma	62	male	26	before
AL4005	screening	adenoma	51	female	21	before
AL4006	screening	adenoma	53	female	38	before
AL4007	screening	neg	65	male	28	before
AL4008	screening	neg	65	male	25	before
AL4009	screening	adenoma	50	male	28	before
AL4010	screening	adenoma	61	female	39	before
AL4011	screening	adenoma	58	male	26	before
AL4012	screening	adenoma	68	male	33	before
AL4013	screening	adenoma	59	male	37	before
AL4014	screening	adenoma	60	female	21	before
AL4015	screening	adenoma	65	female	27	before
AL4016	screening	adenoma	50	male	30	before
AL4017	screening	adenoma	67	female	38	before
AL4018	screening	adenoma	51	male	21	before

AL4019	screening	adenoma	59	male	24	before
AL4020	screening	adenoma	53	male	25	before
AL4021	screening	adenoma	50	male	37	before
AL4022	screening	adenoma	67	male	30	before
AL4023	screening	adenoma	64	male	21	before
AL4024	hospital	cancer	51	male	27	after
AL4025	hospital	cancer	68	male	27	after
AL40262	hospital	cancer	52	male	23	after
AL4027	hospital	cancer	73	male	26	after
AL4028	hospital	cancer	77	male	29	after
AL4029	hospital	cancer	90	female	22	after
AL4030	screening	cancer	67	male	24	before
AL4031	hospital	cancer	76	female	27	after
AL4032	hospital	cancer	69	male	32	after
AL4033	screening	cancer	63	male	27	before
AL4034	hospital	cancer	63	male	27	after
AL4035	hospital	cancer	71	female	29	after
AL4036	screening	neg	69	male	29	before
AL4037	screening	neg	57	female	29	before
AL4038	hospital	cancer	68	male	24	after
AL4039	screening	neg	50	male	32	before
AL4040	screening	neg	57	male	25	before
AL4041	screening	adenoma	68	male	27	before
AL4042	screening	adenoma	63	male	27	before
AL4043	screening	adenoma	64	female	25	before
AL4044	screening	adenoma	52	male	28	before
AL4045	hospital	cancer	66	female	38	after
AL4046	hospital	cancer	57	male	27	after
AL4047	screening	neg	52	male	25	before
AL4048	screening	neg	65	female	25	before
AL4049	screening	adenoma	58	female	39	before
AL4050	screening	adenoma	60	male	30	before
AL4051	hospital	cancer	75	male	25	after
AL4053	hospital	cancer	73	male	26	after
AL4054	hospital	cancer	47	female	29	after
AL4055	screening	cancer	65	male	27	before
AL4056	screening	cancer	63	male	23	before
AL4057	screening	adenoma	58	female	27	before
AL4058	screening	cancer	57	male	21	before
AL4059	hospital	cancer	86	male	23	after
AL4060	screening	cancer	56	female	28	before
AL4061	screening	adenoma	57	female	25	before
AL4062	hospital	cancer	75	female	24	after

AL4063	screening	adenoma	64	male	19	before
AL4064	hospital	cancer	67	male	24	after
AL4065	hospital	cancer	43	male	26	after
AL4066	hospital	cancer	76	male	25	after
AL4067	screening	neg	56	female	24	before
AL4068	screening	neg	54	male	26	before
AL4069	screening	neg	52	female	30	before
AL4070	screening	neg	57	female	24	before
AL4071	screening	neg	52	male	28	before
AL4072	screening	neg	58	female	24	before
AL4073	screening	neg	55	male	29	before
AL4074	screening	neg	54	female	23	before
AL4075	screening	neg	58	female	23	before
AL4076	hospital	cancer	45	male	26	after
AL4077	screening	adenoma	68	male	26	before
AL4078	screening	neg	55	female	25	before
AL4079	screening	neg	54	female	23	before
AL4080	screening	neg	57	female	24	before
AL4081	screening	neg	55	male	26	before
AL4082	screening	neg	50	female	31	before
AL4083	screening	neg	56	male	26	before
AL4084	screening	neg	57	female	23	before
AL4086	screening	neg	51	female	25	before
AL4087	screening	neg	60	male	28	before
AL4088	screening	neg	54	male	27	before
AL4089	screening	neg	56	male	35	before
AL4090	screening	neg	63	male	26	before
AL4091	screening	neg	51	female	30	before
AL4092	screening	neg	51	female	21	before
AL4093	screening	neg	61	female	24	before
AL4095	hospital	cancer	85	male	28	after

Table S3. Thirty-two microbial species selected by 100 times LASSO model, with FDR calculated by ALDEx2.

Specie	1se coef	Times	MWAS	Pr(>|t|).BH
Parvimonas micra	0.313	100	TRUE	9.94E-34
Lachnospira sp000436535	-0.017	100	TRUE	2.26E-12
Faecalibacterium prausnitzii_I	-0.097	100	TRUE	1.15E-11
Agathobacter sp000434275	-0.038	100	TRUE	2.94E-10
Lachnospira sp003537285	-0.005	93	TRUE	2.36E-08
TF01-11 sp003529475	-0.016	100	TRUE	3.58E-08
MGYG-HGUT-03987	0.052	100	TRUE	4.96E-07
CAG-41 sp900066215	-0.017	100	TRUE	6.58E-07
MGYG-HGUT-00605	-0.039	100	TRUE	8.49E-07
Faecalicatena torques	0.02	93	TRUE	8.77E-07
Bacteroides fragilis_A	0.025	93	TRUE	1.52E-06
MGYG-HGUT-00245	-0.037	100	TRUE	2.05E-06
MGYG-HGUT-02304	-0.034	97	TRUE	2.11E-05
MGYG-HGUT-03875	0.041	100	TRUE	2.66E-05
Butyrivibrio_A crossotus	-0.035	100	TRUE	4.02E-05
Blautia_A sp900066205	-0.015	100	TRUE	4.29E-05
Romboutsia timonensis	-0.061	100	TRUE	1.39E-04
UBA11524 sp000437595	-0.021	100	TRUE	1.57E-04
CAG-269 sp000431335	-0.044	100	TRUE	9.48E-04
Sutterella wadsworthensis_A	0.031	100	TRUE	5.69E-03
Anaerotignum sp000436415	0.02	100	TRUE	6.02E-03
MGYG-HGUT-00213	-0.012	95	TRUE	1.23E-02
MGYG-HGUT-00184	-0.052	100	TRUE	1.42E-02
Desulfovibrio sp900319575	0.014	97	FALSE	0.07
Streptococcus thermophilus	-0.047	100	FALSE	0.12
ER4 sp003522105	0.032	100	FALSE	0.34
MGYG-HGUT-00877	0.069	100	FALSE	0.62
CAG-245 sp000435175	-0.01	93	FALSE	0.84
Dialister invisus	0.01	93	FALSE	0.99
Bifidobacterium bifidum	-0.015	97	FALSE	1
Allisonella histaminiformans	0.011	93	FALSE	1
Blautia_A sp000433815	-0.042	97	FALSE	1
Age	0.231			
Sex	0.270			
BMI	-0.090			

Table S4: eggNOG orthologies selected by the predictive model, with effect sizes estimated by ALDEx2 in both the training and the testing datasets. P-value for the testing dataset is also shown.

	Orthology	Training effect	Testing effect	Testing p-value	Annotation
1	COG5498	-0.23	-0.01	8.60E-01	glucan endo-1,4-beta-glucanase activity, C-3 substituted reducing group
2	COG5107	0.14	0.42	2.00E-03	domain of Unknown Function (DUF349)
3	COG4942	-0.01	-0.24	3.90E-02	autolysis
4	COG4887	-0.14	0.03	8.20E-01	protein of unknown function (DUF1847)
5	COG4670	0.14	0.12	3.00E-01	ketone body catabolic process
6	COG3866	0.05	0.44	7.10E-04	pectate lyase activity
7	COG2936	-0.06	-0.1	3.40E-01	dipeptidyl-peptidase activity
8	COG2918	-0.12	-0.3	1.60E-02	glutamate-cysteine ligase activity
9	COG2220	-0.03	-0.13	1.70E-01	N-acetylphosphatidylethanolamine-hydrolysing phospholipas activity
10	COG1925	-0.25	-0.35	1.40E-02	phosphoenolpyruvate-dependent sugar phosphotransferase system
11	COG1803	-0.05	-0.18	1.80E-01	methylglyoxal synthase activity
12	COG1678	0.21	0.32	1.00E-02	ribonucleoside-diphosphate reductase activity
13	COG1501	-0.11	-0.22	6.90E-02	alpha-glucosidase activity
14	COG1489	-0.07	-0.26	3.80E-02	positive regulation of transcription, DNA-templated
15	COG1475	-0.12	-0.32	1.70E-02	chromosome segregation
16	COG1396	-0.14	-0.37	4.30E-03	sequence-specific DNA binding
17	COG1021	0.19	-0.02	8.50E-01	(2,3-dihydroxybenzoyl)adenylate synthase activity
18	COG0818	0.03	-0.27	5.00E-02	undecaprenol kinase activity
19	COG0774	0.22	0.45	7.10E-04	UDP-3-O-[3-hydroxymyristoyl] N-acetylglucosamine deacetylase activity
20	COG0732	-0.08	-0.25	4.40E-02	DNA restriction-modification system
21	COG0682	-0.08	-0.43	2.00E-03	lipoprotein biosynthetic process
22	COG0631	-0.2	-0.5	5.80E-05	protein serine/threonine phosphatase activity
23	COG0515	-0.2	-0.32	1.40E-02	protein kinase activity
24	COG0491	-0.03	-0.37	3.30E-03	hydroxyacylglutathione hydrolase activity
25	COG0142	-0.04	0.11	3.40E-01	isoprenoid biosynthetic process
26	COG0140	-0.19	-0.27	3.60E-02	phosphoribosyl-ATP diphosphatase activity
27	COG0003	0.21	0.16	2.10E-01	ATPase activity
28	2DSTI	0.14	0.03	8.40E-01	*NA*
29	2DMA1	-0.15	-0.33	9.30E-03	*NA*
30	2DBNF	0.41	0.14	3.30E-01	*NA*
31	2DBB0	-0.22	0.03	8.60E-01	L-2-amino-thiazoline-4-carboxylic acid hydrolase
32	2CPUI	-0.29	-0.22	7.40E-02	Stage III sporulation protein AC/AD protein family
33	2BZEU	-0.39	-0.2	1.00E-01	Psort location Cytoplasmic, score
34	2ARFE	0.44	0.17	1.30E-01	Conjugative transposon protein TraO

35	28JU4	0.15	-0.07	5.60E-01	*NA*
36	28HR1	-0.14	-0.26	6.90E-02	Putative amidoligase enzyme
